# Developing Health-Related Indicators of Climate Change: Australian Stakeholder Perspectives

**DOI:** 10.3390/ijerph14050552

**Published:** 2017-05-22

**Authors:** Maryam Navi, Alana Hansen, Monika Nitschke, Scott Hanson-Easey, Dino Pisaniello

**Affiliations:** 1 School of Public Health, The University of Adelaide, Adelaide, SA 5005, Australia; alana.hansen@adelaide.edu.au (A.H.); scott.hanson-easey@adelaide.edu.au (S.H.-E.); dino.pisaniello@adelaide.edu.au (D.P.); 2 South Australia Department for Health and Ageing, Level 1, Citi Centre Building, 11 Hindmarsh Square, Adelaide, SA 5005, Australia; monika.nitschke@health.sa.gov.au

**Keywords:** indicators, climate change, health outcome, vulnerability, stakeholder

## Abstract

Climate-related health indicators are potentially useful for tracking and predicting the adverse public health effects of climate change, identifying vulnerable populations, and monitoring interventions. However, there is a need to understand stakeholders’ perspectives on the identification, development, and utility of such indicators. A qualitative approach was used, comprising semi-structured interviews with key informants and service providers from government and non-government stakeholder organizations in South Australia. Stakeholders saw a need for indicators that could enable the monitoring of health impacts and time trends, vulnerability to climate change, and those which could also be used as communication tools. Four key criteria for utility were identified, namely robust and credible indicators, specificity, data availability, and being able to be spatially represented. The variability of risk factors in different regions, lack of resources, and data and methodological issues were identified as the main barriers to indicator development. This study demonstrates a high level of stakeholder awareness of the health impacts of climate change, and the need for indicators that can inform policy makers regarding interventions.

## 1. Introduction

The progression of climate change is notable in Australia, where the mean surface air temperatures, sea-level rise, and ocean acidification are projected to continue on an upward trajectory [1]. Australia’s climate has warmed by around 1 °C since 1910, and over the past 15 years, the frequency of very warm months has increased five-fold, with more hot days and fewer cool days predicted [1]. 

One of the 17 goals of The United Nations Sustainable Development Goals (SDGs) is to take urgent action to combat climate change and its impacts [2]. Climate change impacts directly and indirectly on human health, as shown in several Australian studies [3,4]. Substantial heat-related morbidity and mortality have been reported in association with extreme heatwaves [5,6,7,8,9,10], and air pollution events due to bushfires and dust storms have been associated with mortality and increased hospitalizations [11,12]. These events are predicted to increase with climate change [13,14,15]. A changing climate can also affect the transmission of climate-sensitive mosquito-borne diseases such as dengue fever and Ross River virus disease [16,17,18], in addition to food-borne diseases such as salmonellosis [19,20,21]. 

Health-related climate change indicators are quantitative measures to monitor the effects of climate change on population health [22] and should be based on a sound link between the exposure to environmental hazards and human health effects [23]. Studies have noted aims to track health effects of climate change using several indicators such as excess morbidity and mortality due to extreme heat; number of injury, death, and mental health outcomes due to extreme weather events including floods and droughts; cases of environmental infectious diseases; and health outcomes related to air pollution and aeroallergens [22,24,25]. These indicators can also be used to assess the effectiveness of public health adaptation strategies and plans. For example, health-related indicators developed in 51 member states of the World Health Organization (WHO) in Europe have had important implications in evaluating heat preparedness planning in the region [26].

At present, in Australia, indicators of environmental change are distinct and separate from indicators of health. The Bureau of Meteorology (BOM) and The Commonwealth Scientific and Industrial Research Organization (CSIRO) are currently using climate data such as temperature and rainfall to monitor the state of the climate and to predict how climate is likely to change in the future [1]. The Australian Institute of Health and Welfare is one of the national organizations which reports on cause-specific health indicators such as asthma and selected infectious diseases [27]. While associations between human health and environmental data have been explored in some cases such as the health impacts of extreme heat waves in 2008, 2009, and 2014 in Australia [28,29,30], a set of regular health-related climate change indicators has yet to be implemented. These indicators would be useful to policymakers and public health authorities to measure and monitor the health effects of climate change over time, identify vulnerable populations, and evaluate the effectiveness of interventions [31]. 

Prior to developing indicators, it is important to define from the start the purpose of the indicators, who will use them, and how they will be used. Therefore, stakeholder involvement at an early stage of indicator development is essential to establish views on the usefulness of and requirements for indicators [32]. The involvement of a broad range of stakeholders from sectors other than health is also necessary, because climate change issues are intersectoral, affecting a multitude of different areas and government departments [33].

The aim of this study is to explore stakeholders’ needs and requirements for measuring and tracking the adverse health effects of climate change and the factors perceived to increase people’s vulnerability to the changing climate. This study, conducted in Adelaide, the capital of the state of South Australia, examines the criteria required to produce robust indicators from the perspective of stakeholders, and the issues they face in developing and using indicators. 

## 2. Materials and Methods

The development of environmental health indicators is complex and requires a deep and nuanced understanding of the needs of stakeholders. A qualitative approach was used in this study to explore the perceptions of the relevant stakeholders, ascertain details about data and information that is essential for stakeholders, and understand the barriers that need to be overcome in developing useful indicators.

### 2.1. Theoretical Perspective

Analysis was undertaken from a critical realist position, as described by Willig [34]. This approach has been extensively used in studies that examine climate-related issues, including mitigation policy and energy technology debates, and relationships between social activities and climate outcomes [35]. Critical realism is a theoretical approach to data analysis concerned with characterizing the nature of reality, whilst acknowledging that a person’s “reality” is bounded by multiple meanings made available to them in the social context [36]. In this way, participants’ views are considered contingent upon locally available knowledge and, thus, analysis does not assume that data constitutes a direct reflection of reality. Rather, it presupposes that interpretation of data is required in order to develop a contextualized understanding of the underlying structures of the phenomenon under examination [34]. In the present study, critical realism aids in understanding stakeholders’ views on the usefulness and development of indicators to measure the health impacts of climate change, backgrounded against the current social context. 

### 2.2. Recruitment

Face-to-face semi-structured interviews were held with key informants and service providers from the state and local government, and non-government organizations in South Australia. Using purposeful sampling [37], potential participants were identified and contacted by the research team who provided information about the study and an invitation to participate. The potential participants were asked about their willingness to participate and were assured of data confidentiality.

Participants included individuals from the health sector, environmental agencies, emergency service organizations, and academics, all working in areas affected by climate change. In total, there were 21 participants from Adelaide, South Australia. 

### 2.3. Data Collection and Analysis

Data collection was undertaken from May 2015 to January 2016. Interviews were conducted at participants’ place of employment and were between 30 min to one hour in duration. All respondents provided informed consent before the interviews proceeded. 

Participants were asked about the need to develop health-related indicators of climate change, data availability, and views of stakeholders about the usefulness of indicators, factors that increase vulnerability or increase resilience to climate change, and issues in indicator development (Table 1).

All interviews were digitally recorded and transcribed using the qualitative analysis software package NVivo 10 (QSR International Pty Ltd., Doncaster, Australia). Transcripts and recordings of the interviews were de-identified to protect confidentiality. 

Methodologically, data were explored inductively using thematic analysis to identify recurring patterns within the data, as proposed by Braun and Clarke [38]. This involved a stepwise process starting with the transcription of the recorded interviews, reading and rereading the text, and noting down initial ideas. Passages of text that displayed similar ideas or concepts were coded and later refined in an iterative process, and finally assigned to particular codes. Codes were then collated into potential themes that were refined and named accordingly. 

### 2.4. Ethics Approval

Ethics approval was granted from the Human Research Ethics Committees at the South Australia Department for Health and Aging and the University of Adelaide (No. HREC/14/SAH/193).

## 3. Results

Of the 21 participants, 14 were from state or local governments, two were consultants, two were academics, and one was from emergency services (Table 2). The expertise and knowledge of the participants were diverse, as organizations and individuals differed in terms of the data they generate or use, services they provide, and their need for indicators. Analysis of the interview data generated five main themes with sub-themes. Themes related to the purpose of using indicators, types of data, criteria for selection of indicators, issues, and alternative indicators (Table 3). 

Participants noted a range of climate change-induced extreme weather events and environmental changes including heat waves, heavy rainfall, droughts, and sea level rise that were potentially linked to adverse effects on human health. They described adverse health effects such as increases in food-borne diseases on hot days, the risk of mosquito-borne diseases that increases with rainfall, and the expansion of standing waters in coastal areas due to sea level rise. 

Participants thought that changes in climate had resulted in hotter weather, were concerned about extreme heat posing a serious risk to the health of vulnerable people, and were aware that the health effects of climate change are not, and will not be, equally distributed. They mentioned factors contributing to vulnerability including: age, needing assistance, ill health, poor English language proficiency, being alone, lack of transport, low level of education, lack of employment, low level household income, financial stress, ethnicity, no access to internet connections, and lack of social connectedness. They also recognized the importance of vulnerability considerations in planning and delivering interventions, and emphasized the need to build community resilience. 

Participants were keen for health-related indicators of climate change to be available and spoke of how they would use indicators, the types of data that would be useful as indicators and the data that are currently available, what makes a good indicator, and issues and barriers to the development of indicators. These issues emerged as the main themes identified from the narratives, as outlined below.

### 3.1. Purpose of Using Indicators

Participants explained the different purposes for which indicators could be used, based on their needs and interests. These included: (i) monitoring and tracking changes in the climate, and the impacts that long and short-term changes might have on human health and the environment; (ii) monitoring disease trends; (iii) measuring adaptation; (iv) evaluating actions taken; and (v) as tools for communication. 

#### 3.1.1. Tracking Changes in the Environment and Monitoring Impacts on People

Participants explained that they use indicators to track environmental changes and monitor impacts that the changes might have on the health of people and the environment. They said they use data that monitors trends over time for temperature, rainfall, soil conditions, droughts, and sea level rise, and these could also be used as ways to mitigate the associated health impacts. This also highlights a gap in data that they need for monitoring the impact of extreme weather events on people and for emergency management.

*“… we can monitor any impacts of climate change whether it would be on how… rainfall might be changing, drying conditions for soil, which has impact on management of open space and reserves, but also so we can monitor the impacts on the community, and obviously health has a huge part of this so that is where this kind of work and developing a really strong indicators set, short term and long term, would be really valuable”*.

(Local government officer 1)

*“I think if you actually did have a set of indicators that really showed this is the impact on health and wellbeing of people from maybe events or slow incremental changes like drought …, I think that actually could be a very powerful tool for actually taking further action in terms of mitigating climate change or adapting to it”*.

(State government officer 1)

Changes in the frequency and intensity of rainfall was mentioned as a good environmental indicator for climate change due to being easily measurable and the known links with some climate-sensitive diseases. An environmental expert also spoke about wind erosion and dust occurring during droughts, and the usefulness of indicators to monitor air quality. An officer in government raised the issue of sea level rise and the expansion of saline water bodies in coastal areas that can consequently increase densities of salinity-tolerant vector mosquitos as *“sea level rise will create more incursion of new breeding sites”*.

#### 3.1.2. Monitoring Disease Trends

It was mentioned that indicators could be used to monitor disease trends and anomalies in the data indicating an abnormally high number of cases warranting public health interventions. Some food borne diseases such as salmonellosis can increase with high temperatures, as can mosquito-borne diseases after heavy rainfall. Interviewees thought that indicators could help monitor case numbers and evaluate the use of interventions. They also mentioned that meteorological indicators such as heavy rainfall could be used as potential predictors of disease outbreaks.

#### 3.1.3. Measuring Adaptation

Participants stated that indicators could be used to measure human adaptation to climate change and how communities function or respond in extreme weather events. Annual or bi-annual reports would help to monitor the progress of climate change adaptation.

A government officer believed that some adaptations to climate change could provide co-benefits for healthier lifestyles. For example, areas of shade or green space in cities can be used not only to measure adaptation to climate change, but also to promote physical activity in the community. 

#### 3.1.4. Evaluation and Assessment

A recurrent theme identified in the data was the use of indicators for the evaluation of public health plans and the effectiveness of programs and actions to reduce the impacts of climate change. Also mentioned was the importance of using indicators for vulnerability assessments and environmental impact assessments in order to provide evidence for the continued funding of successful programs and to assess if adaptation and preventive strategies are successful.

*“In terms of process, I think we need to know what action is happening on the ground to see if it does make an impact on health outcomes and on environment”*.

(State government officer 2)

Indicators have been used in Europe to assess the usefulness of heat-health action plans [26], showing that European countries are partially prepared for the next major heat-wave. For heat health actions plans to be functional and effective, evaluation on a regular basis is necessary [26] and indicators can be useful for this purpose.

#### 3.1.5. Tools for Communication with Policy Makers

Participants stated that indicators can be used as tools to fill communication gaps between scientists and policymakers. They said that using indicators for an evaluation of climate change mitigation and adaptation programs and activities are critical in the current political environment. They expressed views on various ways of presenting information to policymakers and the general public, such as graphs and maps. 

Participants’ views were consistent with recommendations from other studies that the visual presentation of indicators as maps can be effective in raising awareness and informing policy and decision making [39]. Spatial representation of community determinants of heat vulnerability at a national scale in the USA has provided an index for nationwide comparison which has important implications for identifying areas for targeted interventions [40].

### 3.2. Data for Indicators Development

Interviewees mentioned that the types of data collected by organizations include: (i) environmental monitoring data such as air and water quality data; (ii) disease surveillance data; (iii) weather modelling and prediction data; and (iv) survey data. Some organizations did not generate their own data and were dependent on data generated by the Australian Bureau of Statistics (ABS), or other government organizations. Respondents discussed data that were available to them that could be used as health and environmental indicators, and the way that it can be accessed. 

The Environment Protection Authority South Australia, for example, publishes monthly and quarterly air pollution quality summaries and reports online, and daily air quality data over long periods of time that can be made available by request [41]. Disease surveillance data in the form of monthly numbers of notifiable infectious disease cases can be accessed through the National Notifiable Diseases Surveillance System in Australia [42]. Weather modelling and prediction data are provided by BOM and CSIRO [1]. Sixty automatic stations are available in South Australia for collecting weather data, which are available online. In terms of survey data, some local governments undertake local surveys by phone in order to gain subjective self-reported data on different levels of vulnerability and the resilience of communities in terms of an adverse event. It was mentioned that subjective data can reveal how people will function in terms of extreme weather and this important information needs to be collected.

### 3.3. What Is a Good Indicator?

Interviewees spoke of different criteria that robust indicators need to meet. They believed that indicators should be: (1) based on available data; (2) tailored for context; (3) based on a link between environment and diseases; (4) able to be spatially presented; and (5) specific.

#### 3.3.1. Based on Available Data

Participants believed that indicators should be based on available data such as health statistics and environmental data. It is not only easier to use already available data, but also allows the monitoring of issues of concern retrospectively, as well as into the future.

*“I think that would be very important to link the indicators with data that has been collected already. That gives you a very good picture going back as well … but it also gives you more confidence that the data will be collected going in to the future”*.

(State government officer 3)

#### 3.3.2. Tailored for Context

The Australian Bureau of Statistics (ABS) collects vast amounts of data that can be used as indicators in certain contexts. For example, information about the economic and social conditions of people and households within an area can be useful as indicators of vulnerability to climate change. Participants believed that indicators need to be tailored for specific purposes and the current indices are not ideal in all cases. One participant spoke about how they believed Socio-Economic Indexes for Areas (SEIFA) index [43], is not an ideal indicator of vulnerability when applied to country areas, perhaps due to the relatively small heterogeneous populations in large rural areas.

#### 3.3.3. Based on a Link between Environment and Diseases

Credibility is one of the criteria for a robust indicator [44]. Interviewees explained that indicators should be based on a known link between climate and health. In the following quote, the participant discusses rainfall and temperature as environmental indicators and the link with infectious diseases:

*“I think the two of them (rainfall and temperature) make good variables because they are so easy to measure, and so often both are linked to diseases either together or independently… Rainfall and temperature are two of the best indicators”*.

(Academic researcher 1)

Salmonellosis, dengue, and Ross River virus have been mentioned by interviewees of this study and also have been linked with climate change in Australian studies [17,45,46]. However, different climate-sensitive infectious diseases that do not occur in Australia, such as West Nile viruses and Lyme disease, have been suggested as suitable indicators in North America [22,25]. It is therefore important to have indicators that are locally relevant and fit for purpose. 

#### 3.3.4. Spatial Representation of Indicators

Interviewees explained there was a demand for the spatial analysis of data that can be used to produce maps to visually represent several different indicators at once. They thought that data presentation in the form of maps would clearly reveal the areas of change, spatially and temporally, whilst saving many words, graphs, and tables in reports. For example, they can be used by stakeholders to show where flooding is likely to occur, areas of vulnerability, or where certain health outcomes are greatest.

*“One map tells an amazing story compared to what you could, I think that those maps are incredibly powerful for talking with local government councils”*.

(State government officer 1)

*“People find it easy to look at a map and say ok so where do the old people live, where is it going to be flooded …lots of types of vulnerabilities to different risk factors”*.

(Non-government consultant 1)

#### 3.3.5. Specificity of Indicators

Participants’ responses showed that developing a list of indicators might be helpful to stakeholders, but to be practical to use, they need to be specific and fit for purpose. For example, disease data may be required in specific formats such as disease notifications or cases hospitalized. Another example is age as a vulnerability indicator. An older age is a risk factor for heat-related illness, but specific age categories need to be defined as required to be a suitable indicator, as outlined in this quote:

*“what we did first of all, we looked at the, I guess the traditional definitions of vulnerability … we had initially age over 60 and someone said no, people over 60, it’s not over 60 now, it should be over 75 … because people are more healthy and stronger as they are getting older now”*.

(Non-government consultant 1)

### 3.4. Issues and Barriers

Interviewees did not find developing indicators for climate change a straightforward process. A range of issues were noted and are categorized as: climate change is a new complex area; varying risk factors are present in different regions; lack of resources (money, knowledge, and skills) and data; and methodological issues. 

#### 3.4.1. Climate Change Is a New and Complex Area

Respondents spoke of the difficulty in understanding the relationship between climate change and human health and wellbeing, especially for vulnerable populations. Some mentioned that developing indicators for climate change is a new and complex process for them, and interrelationships between factors that impact human health make it difficult to find suitable indicators. They also mentioned that some impacts of climate change may only be seen in the long term.

*“I think it’s difficult to, in a short space of time, to link any changes or any impacts to climate change…Climate change is, as I said, a long-term impact”*.

(State government manager 1)

One of the interviewees suggested that, in response to the issue of the long-term effects of climate change, short term as well as long-term indicators need to be available.

*“I think it is a good idea to have a report annually or every 2 years, that could be quite good if you decide on a very narrow band of the most important indicators, you could have then every ten years a bigger report which would be more meaningful for other indicators, how is it getting worse? Or can we actually adapt? These are really the questions and things that we have not noticed on a yearly level but you can see on a longer term”*.

(State government officer 4)

#### 3.4.2. Variability of Risk Factors in Different Regions

Discrete risk factors are salient in different areas of South Australia due to regional climate variability. While heatwaves occur across the state, there are specific areas prone to sea-level rise, floods, and bushfires. This may cause difficulties in the development and application of indicators. Although South Australian councils work together on climate change adaptation plans across broad regions, issues in local environments are different and councils do not necessarily face the same issues.

*“In different regions, there’s, different climate variables so in terms of climate we had sea-level rise, flooding, and bushfire risk… we also looked at increasing heat. I think sea level rise obviously goes up in some areas, and some areas are bushfire prone while others aren’t”*.

(Non-government consultant 1)

#### 3.4.3. Lack of Resources

Not having knowledgeable people in the planning and vulnerability assessment may lead to some vulnerable communities being overlooked. Respondents claimed that data need to be viewed in the context of local communities and environments. They added that integrating local and scientific knowledge is necessary to make informed decisions.

Respondents mentioned that having a lack of resources limits what they are able to do in terms of their goals and strategic actions. Funding and resources are often insufficient to hire data specialists and analysts. Research was viewed as fulfilling an important role in generating an evidence base and collaboration with research institutes and universities was deemed important.

*“Resources is a really really big barrier and issue for us in terms of what we are able to do, you know often resources don’t meet expectations and there is lot of expectations about what we could be doing and it is already very difficult to match that”*.

(State government officer 1)

#### 3.4.4. Data and Methodological Issues

Data and methodological issues arise in terms of data collection for health-related and environmental indicators of climate change. Issues mentioned include: lack of robust data; data inconsistency and non-comparability due to changes in methods and technology; gaps in data; and not having a central repository of data.

A lack of robust diagnostics and data for some climate-sensitive diseases is a limitation to the development of health-related indicators of climate change. Disease surveillance experts spoke of logistical issues such as laboratory testing for Arboviruses (viruses transmitted by arthropod vectors such as mosquitoes) and the problem of false positives or new testing methods creating inconsistencies in the data. 

Changes in technology over time also cause problems with long-term environmental indicators. An environmental scientist said that current air pollution monitoring instruments are different from the instruments used 30 years ago, which would make comparisons of current data with previous data problematic. Another example is inconsistencies over time in the methods used for flood mapping. Moreover, gaps in the data for some locations impedes the use of current data as indicators and attempts to retrofit data can substantially decrease data accuracy. 

A respondent also alluded to the significance, and yet lack of, subjective data that are needed to measure the community resilience to climate change impacts. They said that it is difficult to gather data on how people perceive changes and develop resilience to extreme weather events and emergencies. An understanding of how individuals and communities prepare for and respond to emergency situations would be useful, as would their perceptions of when weather extremes would exceed coping abilities. It was said that this type of perception data would be useful to stakeholders involved in emergency management planning and service provision.

*“I think a lot of data that we perhaps do not have access to and we simply do not get it, … is that community perception data, so what … does the community need? When do they think it is getting to the point that they cannot function well in a particular climate situation or particular emergency situation? That’s probably something we do not have enough of, we don’t have even systems really to do that well, that would be really valuable to have … it is more that perception data that we are not very good at gathering”*.

(Local government officer 1)

Respondents also mentioned that a central repository of data is essential for more efficient ways to manage and use data as indicators. They are aware of available information, but they did not find it easy to access.

*“We know that government has got lots of information as well, and, there is a barrier there, because there is difficulty in sharing the information, and depositing all the information in one place where everybody can use it”*.

(Local government officer 2)

### 3.5. Alternative Indicators

Respondents provided recommendations on using alternative data that can be helpful in terms of monitoring and tracking changes. Some suggested using environmental indicators as a proxy for health indicators. For example, the surveillance of mosquito populations could be an indication of mosquito-borne pathogens. However, it should be noted that there are many other factors such as the immune status of host populations and socioeconomic conditions that influence disease transmission [47]. Using general practitioner (GP) data as health indicators for morbidity was mentioned by one participant.

*“One type of data that I think is not easy to collect and readily available that could be very informative in detecting not human disease but human pathogens, so what is happening with vector-borne disease at the moment, …, is our ability to detect viruses in the field”*.

(Academic scientist 1)

*“In terms of climate change eventually you have to bring in GP data because there is also lots of information about pre-existing diseases about people who have issues, chronic diseases issues, because you know that … they are prone to be very vulnerable”*.

(State government officer 4)

## 4. Discussion

The aim of this study was to explore stakeholders’ needs and requirements for the development of climate change indicators, their view on robust indicators, and the purposes for which they would use indicators. Stakeholders use indicators for different purposes such as identifying trends over time and monitoring the impact of climate change, taking preventive actions, measuring adaptation, assessing public health plans, and as tools for communication. However, this largely depends on their requirements. 

Our results revealed that stakeholders believed that there would be a tangible impact of climate change on human health and that indicators would be required to measure the impacts. As rising temperature is the environmental indicator most commonly cited in climate change studies [48], participants specifically mentioned increases of heat-related illnesses and death due to climate change. This is supported in the scientific literature which has reported increased heat-related health outcomes as a result of rising temperature [49]. 

Readily available and accessible data for monitoring the impact of climate change are mainly environmental indicators, such as temperature, rainfall, and air pollution data. Health outcome data presently collected in Australia include heat-related mortality and morbidity such as ambulance callouts and hospital admissions, and communicable disease data on food-borne and vector-borne diseases. Similar data are collected in other countries, and in the United Sates [22], Canada [25], and Europe [26,50], excess mortality and morbidity are being used as health indicators of climate change. However, ethics approval is required for accessing health data and resources also need to be made available to undertake relationship analysis to describe links between climate change and human health. The provision of useful environmental health indicators, will require the service of experienced epidemiologists who can undertake quantitative analysis of environment and health associations on a regular basis to capture trends in climate change-related health outcomes. 

For the achievement of Sustainable Development Goals, countries are expected to report progress on the United Nations SDG indicators, and resources should be specifically allocated for this purpose. The indicators include the number of deaths, missing persons, and persons directly affected by disasters, in addition to the proportion of local governments that adopt and implement local disaster risk reduction strategies in line with national disaster risk reduction strategies [2]. The development of environmental health indicators of climate change will aid in monitoring the progress of the SDGs. According to the Australian Bureau of Transport Economics, flood has been the most costly disaster type in Australia, followed by severe storms and cyclones [51]. However, data on human health impacts of floods can be difficult to source, although a study has shown that heavy rainfall and consequent extensive flooding in Queensland in 2010–2011 attributed to 33 deaths [52]. The Insurance Council of Australia provides cost estimates of natural disasters such as death and injuries by hazard type [51], and these could be a potential source of data on injuries and mortality from extreme weather events. 

Indicators provide useful information for local governments when planning for climate change. Preventing development in areas prone to flooding and/or bushfire, and increasing community education and awareness regarding extreme heat, are examples of key priorities considered in the South Australian regional climate change adaptation plans [53]. However, to the authors’ knowledge, records of climate-related adverse events such as flood, bushfire, and storm are not kept in an inclusive database in South Australia. Rather, different organizations and departments keep these records. If these data were managed systematically and centrally, information may be more accessible and useful as indicators of climate change. 

The results of this study have shown that the planning and implementation of interventions often requires an understanding of community resilience to extreme weather events, and it can be difficult to define the questions to ask community members to ascertain perceptions of risk and resilience. A recent study by Bene et al. focused on understanding the factors that influence people’s resilience in fishing communities in Fiji, Ghana, Sri Lanka, and Vietnam that have experienced natural disasters in the past [54]. The authors used a self-assessment questionnaire built around the strategies adopted by households to respond to past floods and tropical storms. Questions focused on how people responded, how they would respond if such events were to happen again in the near future, and how they believed they would be able to recover. These type of questions can be informative and a starting point for local government surveys to gauge community resilience to severe weather events. 

Participants explained that indicators should be: (i) based on available data; (ii) tailored for context; (iii) credible; (iv) represented spatially; and (v) specific. These criteria are similar, but not as wide-ranging, as those identified by other studies for environmental health indicators [23,44] and climate change environmental health indicators [25,55]. Other criteria could also be considered such as cost effectiveness [55] and the quality and integrity of the collected data [25]. 

This study sought to explore the understanding of indicators development within a small group of stakeholders in South Australia. Others, interstate, may have different views or access to different data. Also, as weather and climate characteristics in South Australia can differ between states and regions, and the health burden related to climate change can also vary geographically, not all indicators suggested in this study are necessarily applicable to other areas. Nevertheless, the participants were from several different sectors comprising government, non-government, and academic institutions, thereby providing a wide-ranging picture of stakeholders’ needs for indicators and the issues that they face with the development process. Based on the similarities in the activities, needs, and issues of the participants in other states, the key findings may be useful to policymakers and stakeholders across Australia. Furthermore, given that climate change issues and the related adverse health outcomes have no borders, this study may have an even wider relevance. 

## 5. Conclusions

The study findings have shown the relevance of stakeholder engagement in the process of indicator development to assess their needs and the criteria that are required to ensure that the indicators are robust. The findings show that developing indicators for climate change is not a straightforward process. A range of issues were addressed and included the variability of risk factors for different regions, the potential lack of resources, and data and methodological issues. The four criteria that were of most importance for robust indicators were credibility, specificity, data availability, and being spatially represented. Indicators that seem to be easiest to use and to interpret by stakeholders, and which meet the above criteria, include: environmental indicators such as temperature and rainfall, health outcomes including heat-related mortality and morbidity, and notifications of climate-sensitive diseases. Local and state governments have paid special attention to identifying vulnerable groups; however, current indicators are not always useful in identifying the most vulnerable individuals who may be socially isolated, ill, or disadvantaged for reasons that may not be listed in current databases. The integration of resilience and vulnerability assessments is recommended to provide a more complete story for policy makers and planners in the health and emergency services to aid in the preparation, response, and recovery when facing climate change and future extreme events. This study shows a high level of stakeholders’ awareness on the health impacts of climate change and the need for indicators that can monitor health trends and inform policy making. 

## Figures and Tables

**Table 1:** Interview topic guide.

Question
Can you tell me if your organization collects data regarding extreme weather events, emergencies or natural disasters and if so what type of data this might be?
What is (are) the source(s) of these data and are they routinely collected on a local or national scale? (Secondary question: How are the data collected and is it accessible to researchers?)
Is it just your organization that collects the data or there is a collaboration of organizations?
Are you interested in climate change indicators currently for your work?
How useful do you think this data would be as an indicator to track the progression of climate change, or the health effects of climate change over time, and if so, how?
Are there any data that you think would be useful to collect that might be used as indicators of health outcomes of, or vulnerability to, climate change?
Why do you think you would need them? and what should they look like? How would you use them?
What do you think would be the barriers to collecting these data and their use as indicators?

**Table 2:** Respondent categories by role.

Respondents	Number
State government manager/director	5
State government officer	8
Local government officer	3
Emergency services personnel	1
Non-government consultant	2
Academic	2
Total	21

**Table 3:** Identified themes and sub-themes.

Theme	Sub-Theme
Purpose of using indicators	Tracking and monitoring
	Monitoring disease trend
	Measuring adaptation
	Evaluation and assessment
	Tools for communications with policy makers
Data for indicators development	
A good indicator	Based on available data
	Tailored for context
	Based on a link between environment and diseases
	Spatial representation of indicators
	Specificity of indicators
Issues and barriers	The problem of climate change is a new and complex area
	Variability of risk factors in different regions
	Lack of resources
	Data and methodological issues
